# Tailor-made solvents for pharmaceutical use? Experimental and computational approach for determining solubility in deep eutectic solvents (DES)

**DOI:** 10.1016/j.ijpx.2019.100034

**Published:** 2019-10-31

**Authors:** Henrik Palmelund, Martin P. Andersson, Camilla J. Asgreen, Ben J. Boyd, Jukka Rantanen, Korbinian Löbmann

**Affiliations:** a University of Copenhagen, Department of Pharmacy, Universitetsparken 2, 2100 Copenhagen, Denmark; b Technical University of Denmark, Department of Chemical and Biochemical Engineering, CHEC Research Centre, Søltofts Plads 229, 2800 Kgs. Lyngby, Denmark; c Drug Delivery, Disposition and Dynamics, Monash Institute of Pharmaceutical Sciences, Monash University, Parkville, VIC, Australia

**Keywords:** (COSMO), conductor like screening model, (COSMO-RS), conductor like screening model for real solvents, (DES), deep eutectic solvent, (HPLC), high performance liquid chromatography, (HBA), hydrogen bond acceptor, (HBD), Hydrogen bond donor, (VdW), Van der Waal, Deep eutectic solvents, Pharmaceutical solvents, Excipients, COSMO-RS, Solubility, Drug delivery

## Abstract

A deep eutectic solvent (DES) is a mixture of two or more chemicals that interact via hydrogen bonding and has a melting point far below that of the individual components. DESs have been proposed as alternative solvents for poorly soluble active pharmaceutical ingredients (API). In this study, the solvation capacities of six deep eutectic solvents were compared to water and three conventional pharmaceutical solvents (PEG 300, ethanol and glycerol) for 11 APIs. The experimentally determined solubilities were compared to computational solubilities predicted by the Conductor-like Screening Model for Real Solvents (COSMO-RS). While the conventional pharmaceutical solvents PEG 300 and ethanol were the best solvents for the majority of the studied APIs, API-DES combinations were identified, which exceeded the API solubility found in the conventional pharmaceutical solvents. Furthermore, it was also possible to obtain high solubilities in the DESs relative to water, suggesting DESs to be potential solvents for poorly water soluble APIs. In addition, the relative increase in solubility found in the experimental data could be well predicted ab initio using COSMO-RS. Hence, COSMO-RS may in the future be used to reduce the experimental screening of potential DESs for a given API.

## Graphical abstract

**Figure:**
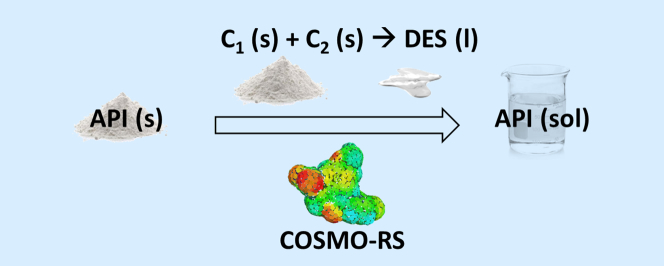

## 1 Introduction

In 2003, a new type of solvent called deep eutectic solvent (DES) with good solvation properties was described by (Abbott et al., 2003). DESs are mixtures of two or more chemicals with a melting point below the melting point of the individual components. DESs are formed by mixing a hydrogen bond acceptor (HBA), typically a quaternary ammonium salt, and a hydrogen bond donor (HBD) (Smith et al., 2014). The first reported DES was a 1:2 molar mixture of choline chloride and urea. Despite melting points of 133 °C and 302 °C for urea and choline chloride, respectively, the mixture was liquid at room temperature with a freezing point of 12 °C (Abbott et al., 2003). After the discovery of the first DES, many combinations of HBA and HBDs such as sugars, polyols, carboxylic acids, and amino acids have been reported to form DESs (Dai et al., 2013; Smith et al., 2014).

The diversity of the starting materials and numbers of combinations allowed the design of numerous DESs with specific physical properties and the ability to dissolve solutes of different nature. The starting materials of DESs are often abundantly found in nature and toxicologically well characterised as non-toxic. DESs have therefore generally been considered non-toxic, biocompatible, and biodegradable. However, a few studies of DES toxicity and biodegradability have reported examples of synergetic toxicologically effects between the individual components when presented as a DES system (Hayyan et al., 2013a; Hayyan et al., 2013b).

As a promising alternative to toxic and environmentally harmful organic solvents, DES have been found applications in the fields of electrodeposition (Abbott et al., 2007; Abbott et al., 2009), biocatalysis (Durand et al., 2013; Zhao et al., 2011; Zhao et al., 2013), extraction of biomaterials, and as medium for chemical synthesis (Gouveia et al., 2016; Handy and Lavender, 2013). A few published studies have suggested DESs as promising solvents for active pharmaceutical ingredients (APIs) with poor aqueous solubility and as an alternative to the conventional pharmaceutical solvents (Li and Lee, 2016; Lu et al., 2016; Morrison et al., 2009). High solubilities of nonsteroidal anti-inflammatory APIs have been reported in DES systems for which the highest solubility (383.4 mg/mL) was measured for ibuprofen in a DES system containing tetrapropylammonium bromide and 1,2-propanediol in a 1:2 molar ratio (Lu et al., 2016). The solubility of the poorly soluble API itraconazole have been reported to be 22 mg/mL in a DES made of choline chloride-malonic acid at a 1:1 molar ratio and 53.6 mg/mL in a DES made of choline chloride-glycolic acid-oxalic acid at a 1:1.6:0.4 molar ratio. These solubilities are 22,000 and 53,600 fold higher than the solubility in water, respectively (Li and Lee, 2016; Morrison et al., 2009). However, none of these studies investigated the solubility of a given API in other conventional pharmaceutical solvents making a true evaluation of the solvation capabilities of DESs difficult. As a result of the vast possibility to combining HBAs with HBDs, the number of potential DESs has been hypothesised to be around 10^6^ (Francisco et al., 2013). The high number of potential DESs request computational methods that can reduce experimental work associated with solubility screening for promising DES-API combinations.

Various computational methods have been developed to predict the solubility of small organic molecules (Bergström and Larsson, 2018). The solubility of an API is determined by the solutes' association with the solvent and its own crystal lattice. Hence, the aqueous solubility of an API can be estimated by the modified general solubility equation from the octanol-water partitioning coefficient (log*P*) and melting point of the solute (Jain and Yalkowsky, 2001). Although this method is simple and based on thermodynamics, it is limited to the aqueous solubility and therefore, not applicable for DESs. The relative solubility (solubility in solvent_1_/solubility in solvent_2_) can be predicted from first principles methods that do not rely on empirical data. These methods do not account for the fusion energy of the solid state but only on the molecular interactions in the liquid phase. The first of its kind was the solubility parameter developed by Hildebrand and Scott (Hildebrand and Scott, 1950), which was later divided into three partial solubility parameters by Hansen (Hansen, 2002). The Hansen solubility parameter is the squared value of partial solubility parameters that describes the contribution of the polar-, dispersion- and hydrogen-bonding-interactions to the solubility (Hansen, 2002). The solubility parameter can be experimentally determined by intrinsic viscosity measurements (Han et al., 2013) or chromatography (Huang, 2004) and estimated by a group contribution method (Van Krevelen and Te Nijenhuis, 2009). If the difference of Hansen Solubility Parameter of solute and the solvent are <0.5 they are estimated to be miscible. However, the difference in Hansen Solubility Parameter cannot be directly translated into an absolute solubility. Another method is the Flory-Huggins solution theory, which is a mathematical model to estimate the miscibility between a solvent and solute by investigating an interaction parameter that represents the free energy of mixing (Flory, 1953). The interaction parameter can be experimentally determined by thermal analysis of mixtures (Rask et al., 2018) or derived from the Hansen Solubility Parameter (Lindvig et al., 2002). Other first principle methods include the purely computational methods molecular dynamics (MD) and Monte Carlo (MC) based simulations as well as the conductor like screening model (COSMO) and COSMO-RS (RS for realistic solvation). MD and MC simulations are explicit models, which can provide valuable insights into molecular solute-solvent interactions (Bergström and Larsson, 2018). These two methods are limited by their high computational costs due to extensive sampling and high degree of freedom for solvent and solute molecules. On the other hand, COSMO and COSMO-RS are much faster computational methods but do not give any explicit information on a molecular level (Klamt, 2005).

The absolute solubility has been computationally predicted by quantitative structural property relationship (QSPR) by modelling molecular descriptors with multi linear regression (Katritzky et al., 1998; Votano et al., 2004), artificial neural network (Huuskonen et al., 1997; Votano et al., 2004), partial least square (Votano et al., 2004), and by deep learning (Lusci et al., 2013) among other modelling approaches. These approaches require experimental data input in order to establish a QSPR that can be used to computationally predict molecules with an unknown solubility. QSPR methods are limited by the chemical space of the training set, and predictions of solute or solvent groups not covered by the training set can be difficult to accurately predict absolute solubilities. However, the advantage of QSPR methods is that they are computational inexpensive and suitable for high throughput screening in early drug development.

Computational methods to determine the crystal structure and energy are fewer and remain a greater scientific challenge. It has been possible to predict the fusion temperature by a QSPR method with a RMSE of 35.1 °C (Bergström et al., 2003). The sixth blind test of organic crystal structure prediction methods revealed that the current computational crystal structure prediction methods have improved remarkably, however, none of the 21 submitted methods was consistently able to predict all the experimental structures (Reilly et al., 2016). Given the lack of QSPR methods for DESs to the best of the authors' knowledge COSMO-RS was deemed to be the best suited computational method for solubility screening of APIs in DESs.

The aim of this study was to first compare the solubility of 11 APIs in six different DESs to their solubility in water and three commonly used conventional pharmaceutical solvents in formulations; and second to evaluate the ability of COSMO-RS as an ab initio predictive screening tool to rank the solvation capabilities of the different solvents.

## 2 Theory of the computational approach

Computational prediction of solvent rank order is useful in order to reduce labour-intensive experimental work and sample consumption during early API development where only limited amount of material is available. Currently, the Conductor-like Screening Model for Real Solvents (COSMO-RS) is considered one of the most accurate ab initio computational methods available for rank ordering of solvents. COSMO-RS is a combination of a conductor-like screening model (COSMO) and statistical thermodynamic treatment of interacting surfaces. Hence, COSMO-RS represent the solvent and solute as small conductor-segments and correct the chemical potential for segment interactions. In the following, the basic concept of COSMO-RS will be described briefly. For a detailed theoretical background of COSMO-RS and COSMO the reader is referred (Eckert and Klamt, 2002) or to a textbook written by (Klamt, 2005).

COSMO-RS considers dispersive, misfit and hydrogen bond (HB) interactions between solute and solvent molecules for calculation of the chemical potential and thus, can be used to estimate the solubility of a solute in a solvent but also other thermodynamic properties.

**Fig. 1:**
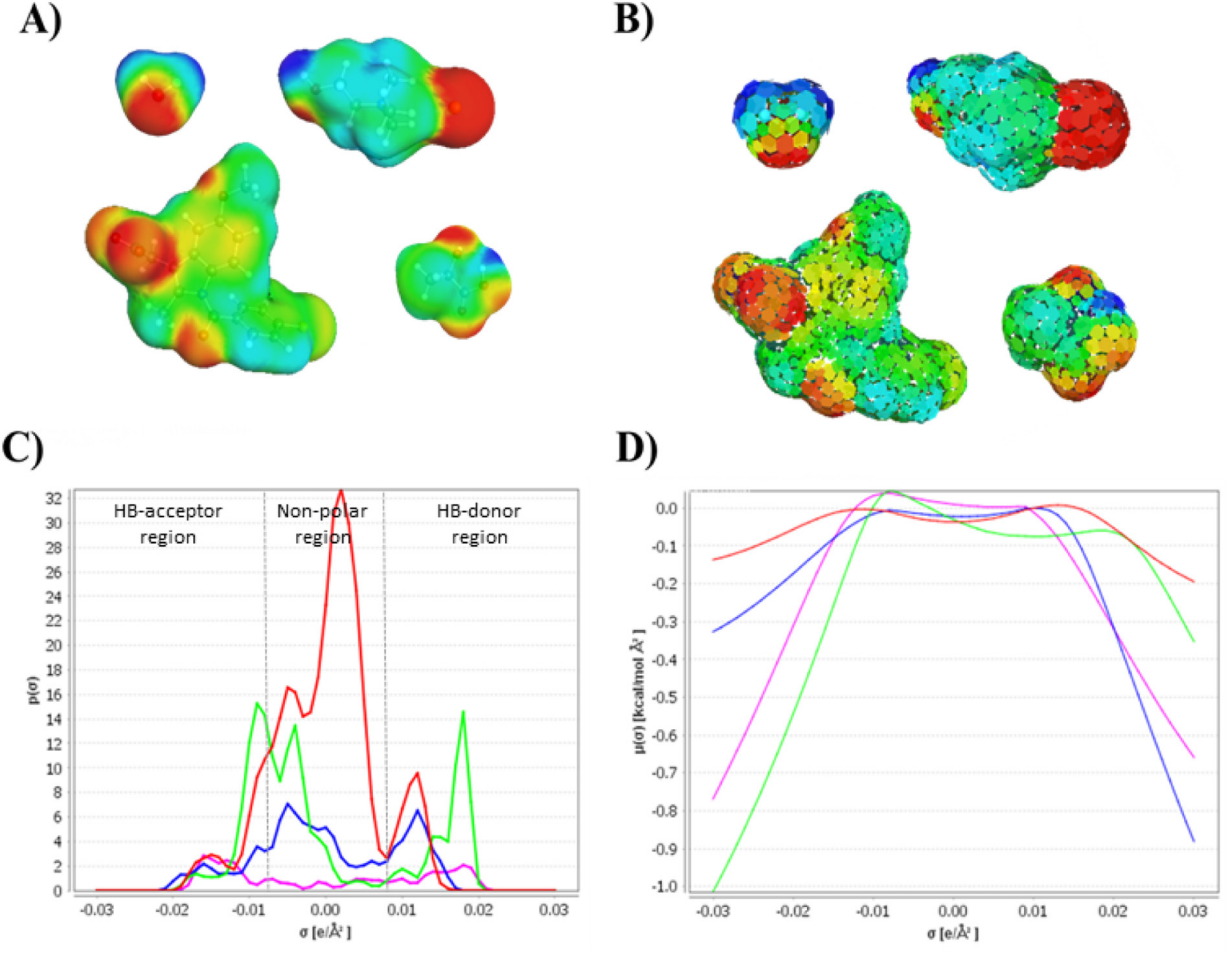
Schematic overview of the computational prediction process by COSMO-RS. A) The screening charge density (*σ*) and three-dimensional structure of for water , choline chloride , indomethacin , and lactic acid . B) A representation of the molecular segmentation of the molecule. C) The *σ*-profile of the solvent and solute. D) From the *σ*-profile the *σ*-potential is determined, which is used to calculate the chemical potential of a solution.

COSMO treats the solvent as a continuum with an infinite permittivity (conductor), which enables calculation of the interaction energy between solvent and solute from the surface charge density (*σ*) of the solute. Hence, a conductor represents the solvent. However, this model is unable to resemble the polar solvents reorientation of permanent dipole moments due to electrostatic interactions with solute molecules. On the other hand, because of the homogenous distribution of the dipole moments of non-polar solvents, the conductor represents the solvent reasonably well to obtain acceptable solvation predictions. COSMO is selected as the starting point for COSMO-RS, i.e. the state of a solute in a perfectly conducting medium (reference state) despite its limitations. Hence, the *σ*-surface of the solute molecules is perfectly screened. The first step in the COSMO-RS prediction is performing the COSMO calculations through a quantum mechanical geometry optimisation in a perfectly conducting medium of all the molecules of interest (both solvents and solutes). The output COSMO-files describing the *σ*-surface of these molecules. Although being time-consuming, the calculations for each molecule can be saved for later use and thus only need to be performed once. The second step is to divide the *σ*-surface into smaller molecular segments, where the VdW-, the misfit-, and HB-interaction energies are calculated from pair wise interaction between molecular surface segments (Fig. 1.B) with a corresponding *σ*-value. The interaction energies are calculated as the energy difference from reference state.

The conductor is assumed to behave as an average van der Waal (VdW)-interaction-partner. Furthermore, it is assumed that in liquid systems, molecular segments will have a VdW-interaction-partner at any time within the VdW-distance. The interaction energy does not differ from the reference state and is consequently neglected in further calculations of the interaction energies. The next interaction to consider is the electrostatic misfit between two segments. If two interacting segments have the same but opposite directed *σ*-value, they have a perfect electrostatic fit and the corresponding energy difference from the reference state will hence be zero. However, this is rarely the case; local electrostatic misfit will occur in the liquid due to thermal fluctuations, steric hindrance or non-availability of the right interaction-partner. The energy (*E*) difference from the reference state is calculated in Eq. (1) as the squared sum of the two interactive segment's *σ* and *σ*′ from the solvent and solute molecule, respectively.

(1) $${∆E}_{\mathrm{misfit}}≅a_{\mathrm{contact}}e_{\mathrm{misfit}}\left(\sigma ,\sigma^{\prime }\right)=a_{\mathrm{contact}}c_{\mathrm{misfit}}{\left(\sigma +\sigma^{\prime }\right)}^{2}$$

where *a* is the surface area (nm^2^) of the interacting segments, *e* is the energy coefficient (kJ/mol/nm^2^) and *c* is a fitting parameter. The last important interaction is the electrostatic attraction between hydrogen atoms bound to an electronegative atom (HB-donor) and an atom bearing a lone pair of electrons (HB-acceptor). For the formation of a HB, the polarity of the HB-acceptor and -donor must overcome a threshold of ±0.0082 e/Å^2^. These relationships are described by Eq. (2), where *σ*_*HB*_^2^ is the required threshold for HB formation and the energy change is given by multiplication of *σ* and *σ*′.

(2) $${∆E}_{\mathrm{HB}}≅a_{\mathrm{contact}}e_{\mathrm{HB}}\left(\sigma ,\sigma^{\prime }\right)=a_{\mathrm{contact}}c_{\mathrm{HB}}\min\left(0,\sigma \sigma^{\prime }-\sigma_{\mathrm{HB}}^{2}\right)$$

The total energy difference from the reference state, caused by electrostatic interactions, is calculated in Eq. (3) as the sum of the misfit and HB energies.

(3) $$\;{∆E}_{\mathrm{int}}≅a_{\mathrm{contact}}e_{\mathrm{int}}\left(\sigma ,\sigma^{\prime }\right)=a_{\mathrm{contact}}\left(e_{\mathrm{misfit}}\left(\sigma ,\sigma^{\prime }\right)+e_{\mathrm{HB}}\left(\sigma ,\sigma^{\prime }\right)\right)$$

Calculation of the surface segments interactions would be extremely complex if the three-dimensional structure of the molecules is retained. However, if the surface is reduced to individual segments (Fig. 1.B) without 3-dimensional geometry restrictions, the calculations of the energy become manageable. Even though neglecting the 3-dimensional structure of the molecule intuitively seems incorrect, it has been shown that this mathematical approach to simplify the calculations can be done without considerable loss of prediction accuracy. A similar approximation is also used in group contribution methods, which is another commonly used approach for ab initio predictions (Van Krevelen and Te Nijenhuis, 2009). The distribution of surface segments according to *σ* is a *σ*-profile and is given by Eq. (4).

(4) $$p_{S}\left(\sigma \right)=\sum_{X_{i}}x_{i}P^{X_{i}}\left(\sigma \right)$$

where *x*_*i*_ is the mole fraction of the individual molecular components *X*_*i*_. An example of an *σ*-profile is seen in Fig. 1.C where the number of segments with an *σ*-value below −0.0082 e/Å^2^ indicates HB-donating ability and *σ*-value above +0.0082 e/Å^2^ indicates HB-accepting ability. The *σ*-profile can be used to calculate the *σ*-potential, *μ*_*s*_(*σ*), which is a characteristic function describing the affinity of the solvent to a surface segment of polarity *σ*. Eq. (5) is solved iteratively usually starting with *μ*_*S*_(*σ*′) = 0.

(5) $$\mu_{S}\left(\sigma \right)=-\frac{\mathrm{kT}}{a_{\mathrm{eff}}}\ln\left[\int d\sigma^{\prime }p_{S}\left(\sigma \prime \right)\times \exp\left\{\frac{-a_{\mathrm{eff}}(e_{\mathrm{int}}\left(\sigma ,\sigma^{\prime }\right)+\mu_{S}\left(\sigma^{\prime }\right)}{\mathrm{kT}}\right\}\right]$$

where *k* is the Boltzmann's constant, *T* the temperature, and *a*_*eff*_ is the effective contact area. An example of a *μ*_*s*_(*σ*) is presented in Fig. 1.D. By using the *μ*_*s*_(*σ*), it is possible to calculate the chemical potential of solute (*X*) in solvent (*S*) by solving Eq. (6).

(6) $$\mu_{S}^{X}=\gamma_{\mathrm{Comb},S}^{X}+\int \mathrm{d\sigma}p^{X}\left(\sigma \right)\mu_{S}\left(\sigma \right)$$

where *p*^*X*^(*σ*) is the *σ*-profile of *X*. The combinational term (*γ*_*Comb*, *S*_^*X*^) corrects for the shape and size effects of *X* and *S*. From the chemical potential, it is then possible to calculate a wide range of physicochemical properties. The solubility can be calculated by Eq. (7).

(7) $$\ln\left(x_{S}\right)=\frac{\left[\mu^{X}-\mu_{S}^{X}-∆G_{\mathrm{fus}}\right]}{\mathrm{RT}\;}$$

where ln(*x*_*s*_) is the logarithmic molar solubility, *μ*^*X*^is the chemical potential of the pure compounds, and *∆G*_*fus*_ is the free energy of fusion. *∆G*_*fus*_ cannot be estimated by the COSMO-RS method and orthogonal computational methods must be used if experimental data is not available. COSMO-RS has also found application in prediction of vapour pressure, partition coefficients, and activity coefficients. In the context of DES, COSMO-RS have been applied for quantitative predictions of phase behaviour and tie-lines for ternary mixtures containing DES in separation of aromatic−aliphatic hydrocarbon mixtures by liquid-liquid extraction (Gouveia et al., 2016). Furthermore, COSMO-RS has been used to predict solid-liquid equilibria of sugar based DES (Silva et al., 2018) and rank-ordering of DESs based on the activity coefficient of rutin in an infinite dilution of rutin and DES (Jelinski and Cysewski, 2018).

## 3 Materials and methods

### 3.1 Materials

The following APIs were purchased from the respective companies: celecoxib (AKScientific, Inc., Union City, CA, USA), cinnarizine and theophylline (Sigma-Aldrich, Steinheim, Germany), flufenamic acid (VWR International, Copenhagen, Denmark), ibuprofen (Chr. Olesen & Co. A/S, Copenhagen, Denmark), indomethacin, naproxen and paracetamol (Fagron Nordic A/S, Copenhagen, Denmark), lidocaine (UNIKEM A/S, Copenhagen, Denmark), and probucol (Tokyo Chemical Industry Co., Ltd., Portland, USA). Aprepitant was kindly donated by Merck (Kenilworth, NJ). Starting materials for the DESs were d-glucose monohydrate (glucoseco), choline chloride, urea, glycerol, betaine, all purchased from Sigma-Aldrich (Steinheim, Germany). dl-Lactic acid was purchased from VWR International (Copenhagen, Denmark). The conventional pharmaceutical solvents, ethanol, glycerol, and polyethylene glycol (PEG) 300 were purchased from Sigma-Aldrich (Steinheim, Germany). Milli-Q water (SG Ultra Clear UV 2002, Evoqua water Technologies LLC, Barsbüttel, Germany) was used for the DES containing water as well as for the analytical work. Hydranal™ and Hydranal™-solvent were bought from VWR International A/S (Copenhagen, Denmark). All chemicals were analytical or Ph.Eur grade and were used as received.

### 3.2 Methods

#### 3.2.1 Preparation of deep eutectic solvents

The DESs listed in Table 1 were selected in order to cover a variety of DES with different HBAs and HBDs. All the mixtures have previously been described to be liquid at room temperature (Abbott et al., 2003; Abbott et al., 2011; Benlebna et al., 2018; Dai et al., 2013; Dai et al., 2015). The DESs were prepared by weighing the desired stoichiometric amounts into a glass vial (with a maximum deviation between theoretic and actual amount of 0.5 wt%). The glass vial was then sealed and the physical mixtures were stirred by a magnetic stirring bar on a hotplate at 50 °C, or at 80 °C (glucose containing DESs).

**Table 1:** Deep eutectic solvents (DES) and molar composition.

DES	Abbreviation	Molar ratio
Choline chloride-urea	CU	1:2
Choline chloride-glycerol	CG	1:2
Choline chloride-lactic acid-water	CLW	1:0.9:0.6
Betaine-glycerol-water	BGW	1:2:1
Choline chloride-glucose-water	CGluW	1:0.4:1
Lactic acid-glucose-water	LGluW	1:0.2:1.2

#### 3.2.2 DES water content determination

The water content was determined using automated Karl Fischer (KF) titration (870 KF Titrino, Mettler Toledo, Glostrup, Denmark) with Hydranal™ in Hydranal™-solvent at room temperature. The Hydranal™-solvent was dried by means of titration. When a steady baseline was achieved, a given DES was added to Hydranal™-solvent by a syringe; and the solution was thereafter stirred for 5 min to ensure a homogeneous solution. The exact amount of DES was determined by weighing the syringe before and after addition to the Hydranal™-solvent. The titer was determined by sodium tartrate dihydrate (15.7 wt% water) according to the European Pharmacopeia (2.5.12; method A).

#### 3.2.3 Experimental solubility

The solubility of the APIs in water, DESs and conventional pharmaceutical solvents was determined by addition of an excess amount of solid API to approximately 2 g of DES. Agitation was applied by a magnetic stirring bar in a sealed glass vial for 24 h at room temperature. The solution was subsequently separated from the excess solid by centrifugation at 15,000 rpm for 30 min, the supernatant was hereafter centrifuged for an additional 30 min. If the excess solid separated towards the top, the solid was removed and the sample centrifuged for an additional 5 min; this step was repeated until no solid material was floating on top of the solution. Excess solid naproxen was removed from glycerol by filtration (pore size 0.22 μm, Q-Max® NY, Frisnette, Denmark), as separation of solid and liquid was not obtained after centrifugation. The concentration of the API dissolved in the DESs was quantified by UV-spectroscopy or high performance liquid chromatography (HPLC) with UV detection. A known amount of DES-API solution was diluted in a sufficient volume of ethanol and water (1:1) for UV-spectroscopy and with mobile phase for HPLC analysis to ensure that a solution without precipitates of API and/or DES components. The solutions were visually inspected for precipitates.

#### 3.2.4 High performance liquid chromatography (HPLC)

The chemical quantification was performed on Dionex HPLC system (Dionex™, CA, USA). A known amount of DES-API solution was diluted in mobile phase for HPLC analysis. All HPLC samples were injected (2–50 μL) on a Kinetex® 5 μm EVO C18 100 LC column 100 × 4.6 mm (Phenomenex, Værløse, Denmark). The HPLC were equipped with an ASI-100™ automated sample injector and P680 HPLC pump (Dionex™, CA, United States) and a PDA-100 (Thermo Fisher Scientific, Waltham, MA, USA) for UV-detection. The chromatograms were analysed using Thermo Scientific Dionex Chromeleon 7 Chromatography Data System software (Thermo Fisher Scientific, Waltham, MA, USA).

Standard curves were constructed by serial dilution of stock solution in mobile phase. All samples were within the concentration range of the standard curve (R^2^ > 0.99) and above the limit of quantification. For all methods (see Table 2) a constant flow rate of 1 mL/min were applied.

**Table 2:** High performance liquid chromatography method details.

API	Aqueous mobile phase	Organic mobile phase	Composition (v/v)	Detection wavelength (nm)	Retention time
Indomethacin	Water + 0.05% trifluoroacetic acid	Acetonitrile + 0.05% trifluoroacetic acid	50:50	264	3.7
Cinnarizine	5 mM phosphate buffer, pH 10	Acetonitrile	30:70	220	5.1
Probucol	Water	Acetonitrile	90:10	200	6.2
Flufenamic acid	0.1% phosphoric acid	Acetonitrile	45:55	200	8.3
Celecoxib	Water	Acetonitrile	60:40	254	2.4
Aprepitant	25 mM ammonium acetate	Acetonitrile	45:55	220	2.4

#### 3.2.5 UV-spectroscopy

A known amount of DES-API solution was diluted in a sufficient volume of ethanol and water (1:1 v/v).The concentration of the API dissolved in the DESs was quantified by UV-spectroscopy (UV-1800, Shimadzu, Japan). All samples were within the concentration range of the standard curve (R^2^ > 0.99) and above the limit of quantification. The detection wavelength for ibuprofen, lidocaine, naproxen, paracetamol, and theophylline were 260, 253, 275, 260, and 272 nm respectively.

#### 3.2.6 Computational solubility predictions

The COSMO-RS method was used for computational predictions of the relative solubility of the APIs in DESs. COSMO files with a TZVP quantum chemical level were obtained from Turbomole version 7.3 (Ahlrichs et al., 1989). The computational solubility predictions were performed by COSMOtherm (COSMOlogic GmbH & Co. KG, Leverkusen, Germany; Eckert and Klamt, 2002) with the TZVP_18.ctd parameterisation. The solubility was predicted by the iterative method both in water, ethanol, and in the DESs by selecting a solvent mixture with the molar compositions equal to the DESs. All solvent - and API molecules were treated as unionised and only present in the most stable conformation. COSMO-RS is a fluid phase thermodynamic model, and for that reason, computational predictions of absolute solubilities require information about the free energy of fusion for each component. To avoid input of experimental values the fusion energies (*∆G*_*fus*_) are estimated by a group contribution method in the COSMO*therm* software. However, this estimation of the fusion energy compromises the solubility predictions. Hence, in order to circumvent the challenges of predicting the fusion energies, the solubility was predicted as a relative solubility rather than an absolute solubility. Since experimental solubilities for aprepitant and probucol in water could not be determined because of their poor aqueous solubility, ethanol was used as a reference for these two APIs.

## 4 Results

### 4.1 Water content of the deep eutectic solvents

The water content of the DESs was determined using Karl Fischer. It was possible to analyse four out of six DESs; the remaining two DESs systems contained glucose and could not be analysed with the current KF solvents. The DES contained following percentage of water: 0.2% w/w in CG, 0.1% w/w in CU, 6.1% w/w in CLW and 5.0% w/w for BGW. These results are in agreement with the starting composition of the DESs. While the majority of the DES had absorbed moisture during handling, BGW and CLW had lost water during the preparation of the DES. Thermogravimetric methods were also applied but the results overestimated the water content due to evaporation of the DES components.

### 4.2 Experimental solubility

**Fig. 2:**
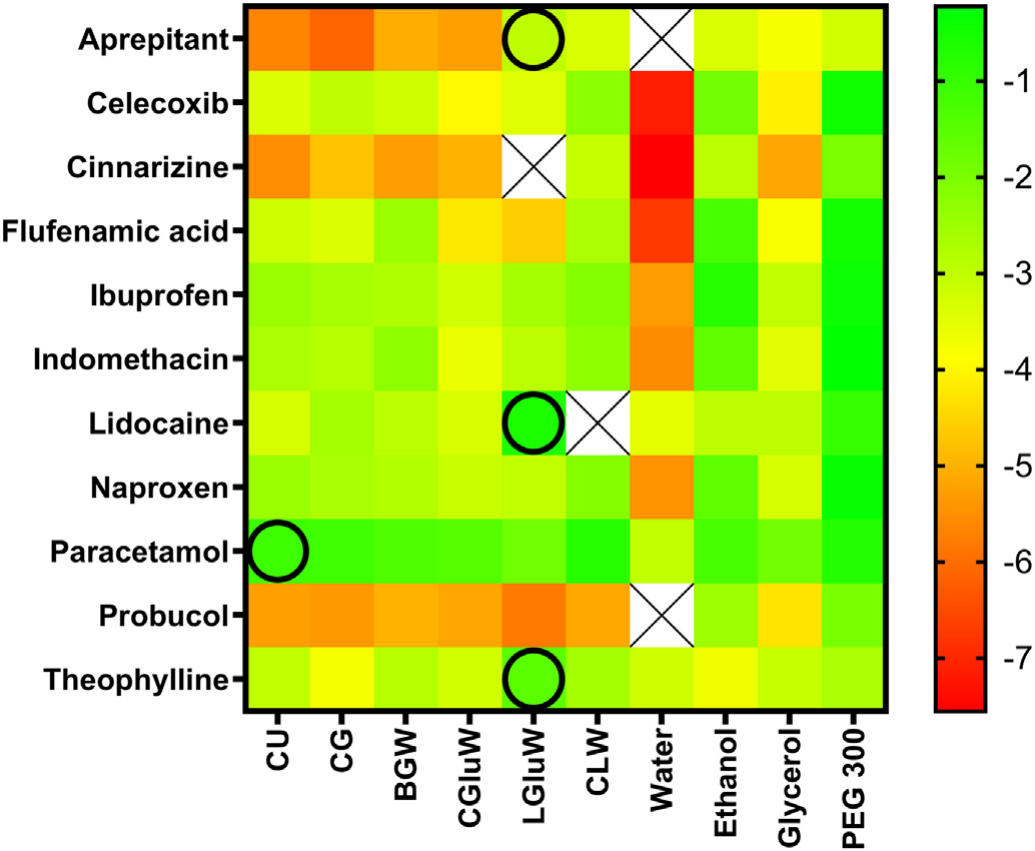
Graphical representation of the experimental logarithmic solubility (mole/mole) of the APIs in the DESs, water, and conventional solvents. The colours green and red indicate high and low solubility, respectively. The solubility of aprepitant and probucol was below the limit of quantification in water and the missing data is indicated by a cross. The circles indicate API-DES combinations that outperform conventional pharmaceutical solvents in a w/w solubility. (For interpretation of the references to colour in this figure legend, the reader is referred to the web version of this article.)

The solubilities of the APIs in water, conventional pharmaceutical solvents, and DESs are presented in Fig. 2, Table 3, and Fig. S1. The APIs covered a broad range of solubilities, where some APIs showed a narrow solubility span (e.g. paracetamol and theophylline) and other APIs a broad solubility span (e.g. celecoxib and flufenamic acid) in the studied solvents. The solubility of the APIs in the majority of DESs and conventional pharmaceutical solvents were substantially higher compared to the solubility in water. When comparing the solubilities of the 11 APIs in the three conventional solvents, PEG 300 and ethanol were better solvents than glycerol (Fig. 2). With respect to the studied DESs, the solvation capacity was generally in a similar range as the conventional pharmaceutical solvents and very comparable to glycerol.

**Table 3:** Solubility of APIs after 24 h of agitation at room temperature (n = 3).

Solvent	Solubility (mg/g ± SD)									
	CU	CG	CLW	BGW	LGW	CGW	Ethanol	Glycerol	PEG 300	Water
Naproxen	9.5 ± 0.1	4.4 ± 0.1	14.2 ± 0.1	4.6 ± 0.2	1.85 ± 0.08	2.89 ± 0.06	110 ± 6	1.25 ± 0.05	412 ± 11	0.0452 ± 0.0005
Paracetamol	123 ± 4	94.8 ± 0.8	200.5 ± 6	80 ± 6	26.2 ± 2	54.0 ± 2	155 ± 3	20.9 ± 0.3	102 ± 1	7.9 ± 0.2
Ibuprofen	9 ± 2	4.5 ± 0.8	14.0 ± 0.5	4.9 ± 0.2	6.1 ± 0.2	1.30 ± 0.1	467 ± 18	2.3 ± 0.1	304 ± 9	0.055 ± 0.004
Flufenamic acid	2.19 ± 0.05	1.05 ± 0.05	5.02 ± 0.06	11.8 ± 1.0	0.085 ± 0.003	0.176 ± 0.006	266 ± 6	0.45 ± 0.06	303 ± 15	0.0029 ± 0.0001
Indomethacin	8.4 ± 0.1	5.0 ± 0.2	15.9 ± 0.9	23.0 ± 0.6	5.06 ± 0.05	0.95 ± 0.00	160 ± 8	1.34 ± 0.08	632 ± 23	0.055 ± 0.004
Lidocaine	1.36 ± 0.06	5.77 ± 0.07	134.7 ± 2	3.48 ± 0.02	459 ± 15^a^	1.2 ± 0.1	20.5 ± 0.7	10.6 ± 0.2	307 ± 3	3.80 ± 0.05
Theophylline	2.2 ± 0.2	0.28 ± 0.03	4.1 ± 0.1	3.42 ± 0.04	63.5 ± 0.5	1.08 ± 0.03	0.81 ± 0.02	1.66 ± 0.07	1.23 ± 0.09	6.4 ± 0.1
Celecoxib	1.78 ± 0.03	3.86 ± 0.02	18.6 ± 0.0	3.25 ± 0.04	1.81 ± 0.00	0.40 ± 0.02	100 ± 5	0.33 ± 0.03	395 ± 36	0.00148 ± 0.00007
Aprepitant	0.0133 ± 0.0003	0.0038 ± 0.009	2.03 ± 0.3	0.058 ± 0.009	7.2 ± 0.1	0.031 ± 0.02	4.9 ± 0.3	0.973 ± 0.06	1.0 ± 0.2	Below LOQ
Probucol	0.0318 ± 0.001	0.02 ± 0.01	0.03 ± 0.01	0.067 ± 0.009	0.010 ± 0.004	0.034 ± 0.03	36 ± 2	0.3 ± 0.2	19.0 ± 0.2	Below LOQ
Cinnarizine	0.013 ± 0.004	0.06 ± 0.02	2.8 ± 0.5	0.04 ± 0.03	b	0.038 ± 0.03	9.5 ± 0.5	0.03 ± 0.01	12.4 ± 0.3	0.0006 ± 0.0001

a Turns very viscous during dissolution of lidocaine disabling stirring of the solution.

b Phase separation of the DES system was observed after dissolution of API.

For four out of 11 APIs, the solubility was higher in a DES than any of the conventional pharmaceutical solvents. Lidocaine had the highest solubility in LGluW (469.4 mg/g), paracetamol in CLW (200.5 mg/g), theophylline in LGluW (63.5 mg/g), and aprepitant in LGluW (7.22 mg/g). The LGluW system contained the largest molar ratio of lactic acid of the DES in the study. As lactic acid is liquid at room temperature the solubility of lidocaine, theophylline, and aprepitant was measured in lactic acid alone. Lidocaine was found to form a very viscous liquid mixture in a 1:1 molar ratio with lactic acid at room temperature. The solubility of theophylline and aprepitant was measured to be 116.4 ± 2.7 and 83.5 ± 22.8 mg/g in lactic acid, which is 1.8 and 12-fold higher than in LGluW, respectively. Thus, none of these three APIs were more soluble in LGluW than in lactic acid alone. On the other hand, paracetamol had a solubility of 96.5 ± 5.5 mg/g in lactic acid. Thus, despite the limited data set, it was possible to find one API-DES combination (paracetamol-CLW) for which the API solubility was higher than in any single component solvent. High solubilities of paracetamol were also obtained in CU, CG and BGW with a solubility of 122, 94.8, and 80.3 mg/g, respectively. The solubility of paracetamol, ibuprofen, and naproxen in CU, CG, and CLW has previously been reported by (Lu et al., 2016) along with the solubility of other non-steroidal anti-inflammatory APIs in various DES systems. The reported solubilities of these nine API-DES combinations are consistent with the solubilities obtained in this study (see Table S1). No signs of chemical degradation could be observed based on the chemical analysis.

### 4.3 Computational solubility

**Fig. 3:**
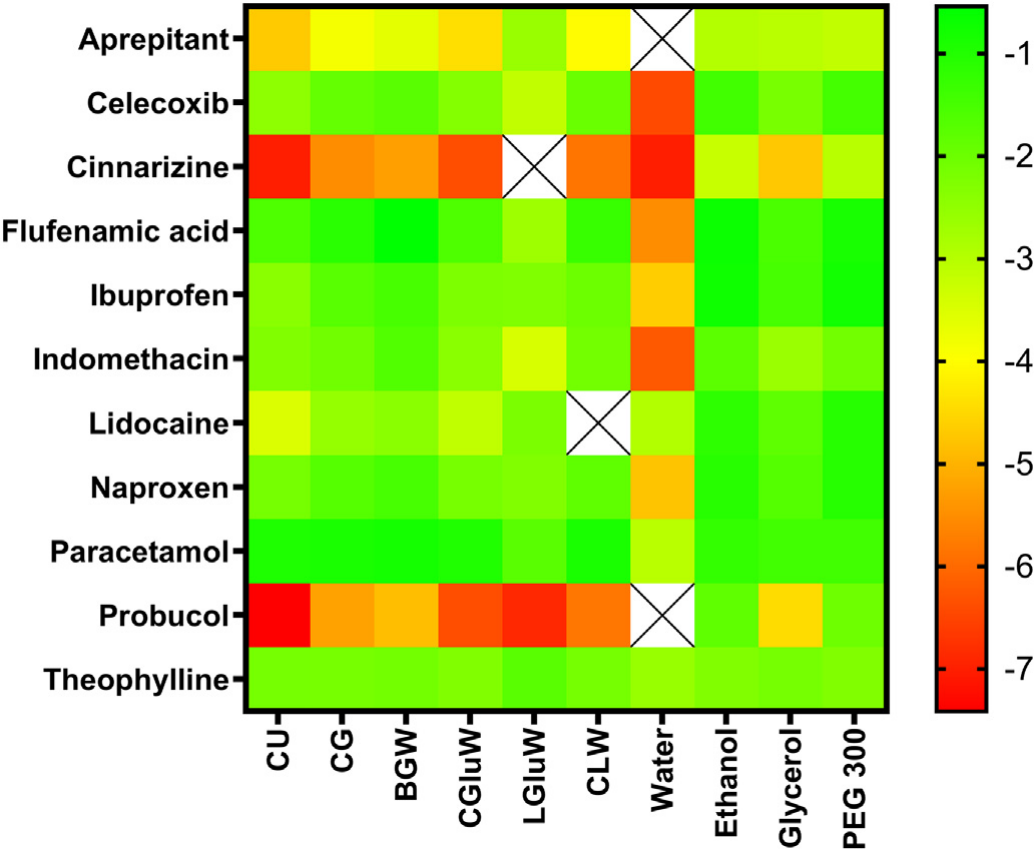
Graphical representation of the COSMO-RS predicted logarithmic solubility (mole/mole) of the APIs in the DESs, water, and conventional solvents. The colours green and red indicate high and low solubility, respectively. Missing data is indicated by crosses. (For interpretation of the references to colour in this figure legend, the reader is referred to the web version of this article.)

**Fig. 4:**
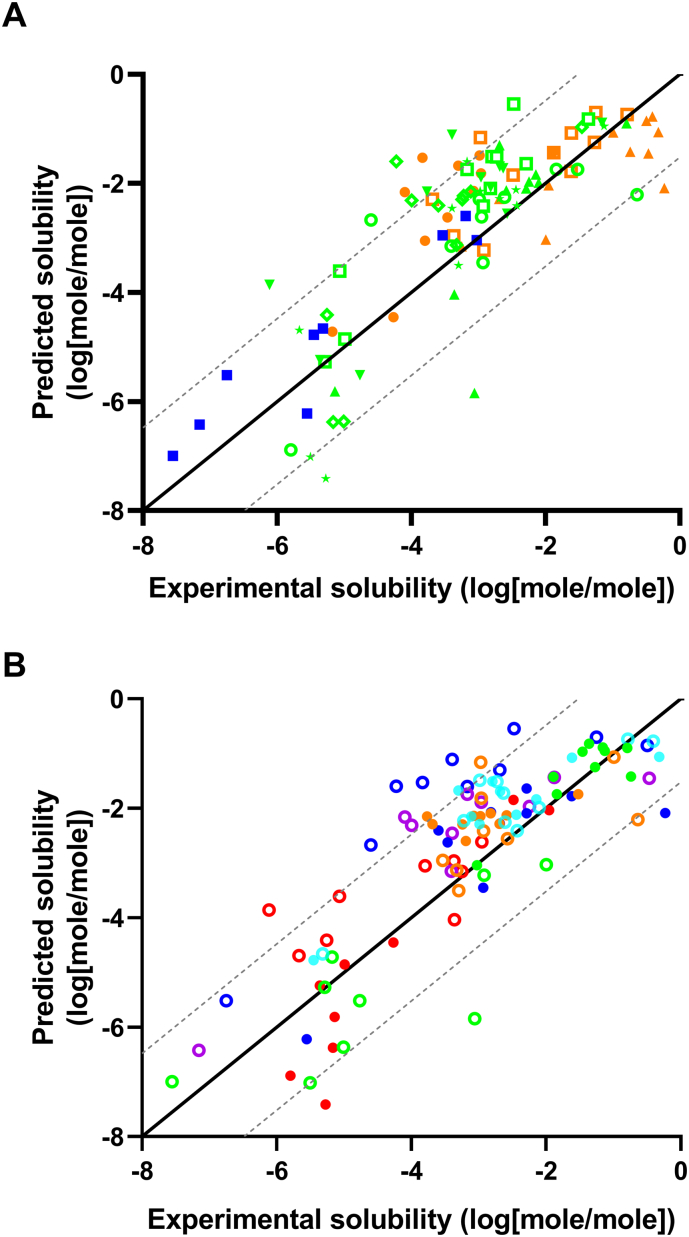
A) Predicted vs. experimental solubility for all 11 APIs in water, glycerol, ethanol, PEG 300 and the six different DESs (CU, CG, BGW, CGluW, LGluW, and CLW). The black solid line illustrates the ideal fit and the grey dotted line represents the expected 0.95 confidence interval of the computational predictions. Slope of best linear fit = 0.90, intercept = 0.053, R^2^ = 0.68. RMSE = 1.044. B) Predicted vs. experimental solubility plotted according to API. Aprepitant, celecoxib, cinnarizine, flufenamic acid, ibuprofen, indomethacin, lidocaine, naproxen, paracetamol, probucol, and theophylline.

The solubility of the APIs was predicted in the molar composition of the DES as well as in glycerol, ethanol, PEG 300, and water. The predicted solubilities of the different APIs grouped similarly to the experimental data, as indicated by the similarity of Fig. 2, Fig. 3 (the numeric values can be found in Figs. S1 and S2). This is further supported by plotting (R^2^ = 0.68; Fig. 4) the experimental solubility data against the computationally predicted data. For solvents with a considerable solubility difference it was possible to rank-order the solvents. However, for solvents with a roughly similar solubility it was not possible to obtain a correct rank-order.

**Fig. 5:**
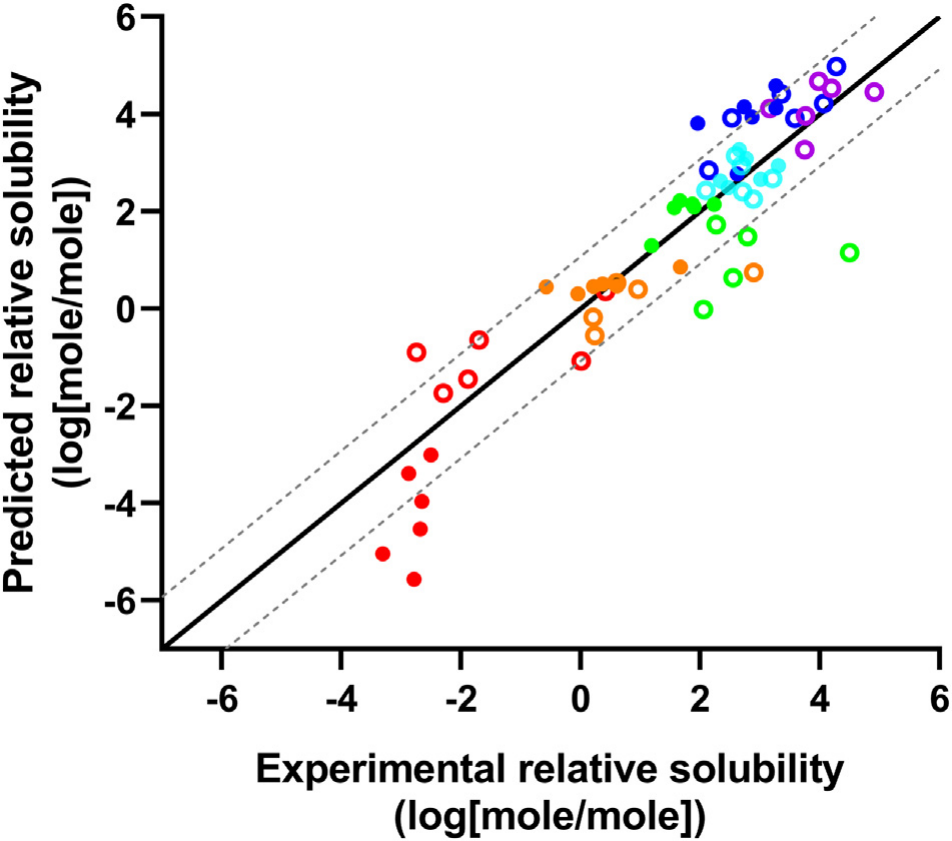
The experimental solubility and COSMO-RS predicted solubility in DES relative to water (* or ethanol). The black solid line illustrates the ideal fit and the grey dotted line represents the expected 0.95 confidence interval of the computational predictions. Slope of best linear fit = 1.07, intercept = −0.18, R^2^ = 0.82, RMSE = 1.042. Aprepitant*, celecoxib, cinnarizine, flufenamic acid, ibuprofen, indomethacin, lidocaine, naproxen, paracetamol, probucol*, and theophylline.

In order to improve the predictive capability of the COSMO model, the relative solubilities were compared, which allows avoiding the challenges associated with estimating the free energy of fusion for each compound. The systematic shifts for some APIs (aprepitant, cinnarizine, flufenamic acid, and probucol) that were observed in Fig. 4 were to a great extent eliminated in Fig. 5, which indicate that this systemic shift have been due to the estimation of free energy of fusion. The solubilities of the APIs in DESs relative to water provide insights into the enabling potential of the DESs for poorly water soluble APIs. For this reason, combined with the availability and low costs, water was chosen as the primary reference solvent. For probucol and aprepitant it was not possible to experimentally determine the solubility in water and ethanol was for that reason used as an alternative reference solvent. A comparison of the experimental and predicted relative solubility of the investigated APIs is presented in Fig. 5. It can be seen that the computational solubility prediction improved significantly (R^2^ = 0.83).

## 5 Discussion

### 5.1 Experimental solubility

The solubility results demonstrated the possibility to obtain high solubilities of APIs in DES systems; however, the results also showed that the solubility for most APIs was higher in the conventional pharmaceutical solvents. Although conventional pharmaceutical solvents generally have better solvation properties than DESs some of the conventional pharmaceutical solvents also have clear limitations to their use due to toxicity and/or religious non-acceptance such as ethanol. The solubility of paracetamol was 1.3-, 2.0-, 2.1-, and 9.7-fold higher in CLW (200 mg/g) than in ethanol, glycerol, lactic acid, and PEG 300, respectively. Of all investigated APIs and solvents, celecoxib showed the largest increase in solubility relative to water, which was 4.91 log units (mole/mole) higher in CLW (18.3 ± 0.0 mg/g) compared to water (0.00148 ± 0.00007 mg/g). These results demonstrate that it is possible to obtain solubilities of APIs in DESs that exceed the solubility in conventional pharmaceutical solvents.

The solubility of the APIs in DESs was for the majority of combinations considerably higher than in water and to some degree comparable to the conventional pharmaceutical solvents. The hydrophilic nature of the individual components in the selected DESs, may have been a limiting factor for the solubility for some of the APIs. With potentially 10^6^ different DESs it may be possible to obtain higher solubilities with other DESs; which are made by components with the ideal polarity and functional groups to facilitate a higher solubility. It may also be possible to optimising the molar composition of the DESs as demonstrated by (Lu et al., 2016).

Based on this study, DESs present a potential alternative to conventional pharmaceutical solvents for poorly soluble APIs. DESs provide the opportunity to access solvation properties of otherwise solid components and to design solvents that are optimal for a specific API or formulation. However, the numerous potential combinations of components and molar ratios emphasise the need for computational methods that can reduce the amount of experimental work required.

### 5.2 Computational solubility predictions

Experimental solubility measurements are time and labour consuming. Computational methods would therefore be highly valuable to identify the most promising DESs for a given API. The computational approach based on the COSMO-RS predictions of absolute solubilities indicates the potential of this method for a rough selection of promising DES-API combinations (Fig. 4). A better approach to avoid the challenges of predicting the energy of fusion was to predict the relative solubility (Fig. 5). The plot demonstrates that COSMO-RS is able to guide the selection of API-DES combinations in order to potentially ease the time-consuming experimental solubility screening associated with identification of promising API-DES combinations. Furthermore, the best linear fit slopes of 0.90 and 1.07 in Fig. 4, Fig. 5, respectively, demonstrated that the solvation of APIs in DESs roughly follow the existing fluid phase thermodynamic theory that COSMO-RS is based on.

Computational prediction of the solubility of APIs in conventional pharmaceutical solvents by COSMO-RS has previously been reported by (Pozarska et al., 2013) where the predictions showed the same rank-order for 5 out of 7 APIs between the COSMO-RS predicted and experimental rank-order for the majority of the 16 pure solvents such as: Ethanol, PEG 400, dimethyl sulfoxide, benzyl alcohol, oleic acid, etc. The results from this study further supports that COSMO-RS is a useful tool to assess a solvent ranking for a given API, including also multi-component solvents such as DESs. This may in future help to ease the selection of the right solvent with the most pronounced solubilisation increase. While the computational predictions can reduce the experimental workload to select the best solvents among a set of given solvents, the computational predictions cannot replace the experimental measurements of the absolute solubilities in these solvents.

## 6 Conclusion

Overall, conventional pharmaceutical solvents performed generally equivalent to or better than the investigated DESs in solvating the 11 APIs of this study. However, for four out of 11 APIs, the solubility was higher in a DES than in any of the conventional pharmaceutical solvents. When testing the solubility in the liquid components of the DESs, we successfully identified one API-DES combination in which the API solubility was higher than in any conventional pharmaceutical solvent or single component of the DESs. The solubility of paracetamol was 1.3, 2.0-, 2.1-, and 9.7-fold higher in a DES containing choline chloride, lactic acid and water (200 mg/g), compared to ethanol, glycerol, lactic acid, and PEG 300, respectively. The highest solubility difference between water and DES was measured for celecoxib where the solubility was 4.91 log units (mole/mole) higher in DES (18.3 mg/g) compared to water (0.00148 mg/g). Hence, DESs are potential carriers for poorly soluble APIs as they showed high relative solubilities of poorly soluble APIs. Given the limited number of DES tested in this study compared to the numerous potential DESs available, other DES compositions with even higher solvation capacities could potentially exist. Nevertheless, finding such a feasible DES-API combination can be very labour intensive and predictive tools are therefore essential for a fast feasibility assessment of DESs as alternatives to conventional pharmaceutical solvents. The results from this study showed that the relative solvation capacities could be rank-ordered (R^2^ = 0.82) ab initio using COSMO-RS calculations. Hence, COSMO-RS may in the future be used to reduce the experimental solvent screening for identification of the best DES for a given API, particularly in expanded systematic studies with a large number of DES combinations.

## Declaration of competing interest

The authors have no conflict of interest to declare.

## Appendix A Supplementary data

Supplementary material Image 37
