# Supplementary material for: Tailor-made solvents for pharmaceutical use? Experimental and computational approach for determining solubility in deep eutectic solvents (DES)
Source: Int J Pharm X. 2019 Oct 31;1:100034. doi: 10.1016/j.ijpx.2019.100034 (PMC6977171; doi:10.1016/j.ijpx.2019.100034)
Supplement: Supplementary file 1 — Supplementary material [file mmc1.pdf]

Supporting information

Results

Experimental solubility data

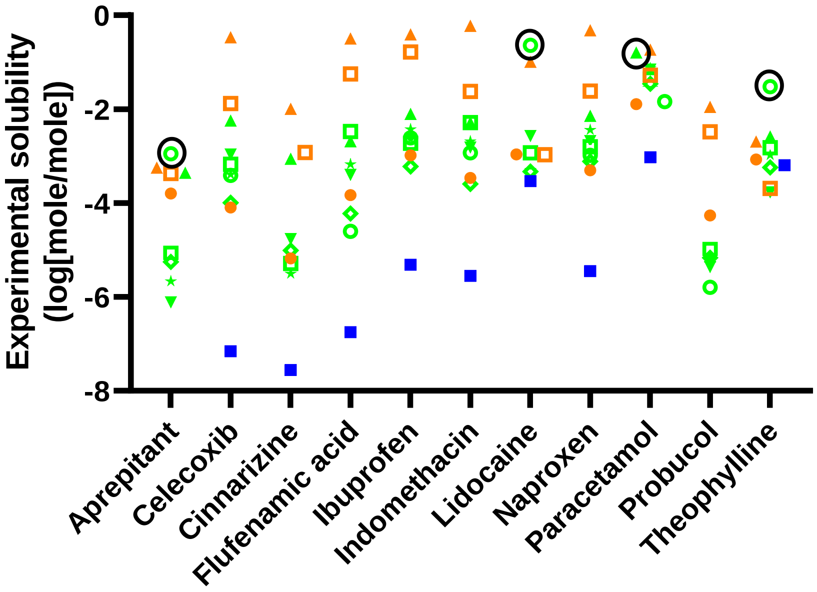

Figure S1. The experimental solubilities of the APIs in in in ■water, ●glycerol, □ethanol, ▲PEG 300 and the six different DESs (★CU, ▼CG, □BGW, ◇CGluW, ○LGluW, and ▲CLW). The solubility of aprepitant and probucol was below the limit of quantification in water. The circles indicate API-DES combinations that outperform conventional pharmaceutical solvents in a w/w solubility.

Table S1: Comparison of solubility of paracetamol, ibuprofen, and naproxen in three DESs (CU, CG, and CLW) to the solubilities reported by Lu et al., 2016.

API	DES	Solubility	
		(mg/g)	Lu et al.. 2016 (mg/mL)
Paracetamol	CU	123 ± 4	169.7 ± 1.98
	CG	94.8 ± 0.8	140.3 ± 2.58
	CLW	200.5 ± 6	254.0 ± 1.84
ibuprofen	CU	9 ± 2	4.500 ± 0.01
	CG	4.5 ± 0.8	3.820 ± 0.03
	CLW	14.0 ± 0.5	17.95 ± 0.25
naproxen	CU	9.5 ± 0.1	15.46 ± 0.11
	CG	4.4 ± 0.1	3.690 ± 0.04
	CLW	14.2 ± 0.1	12.95 ± 0.07

Computational solubility data

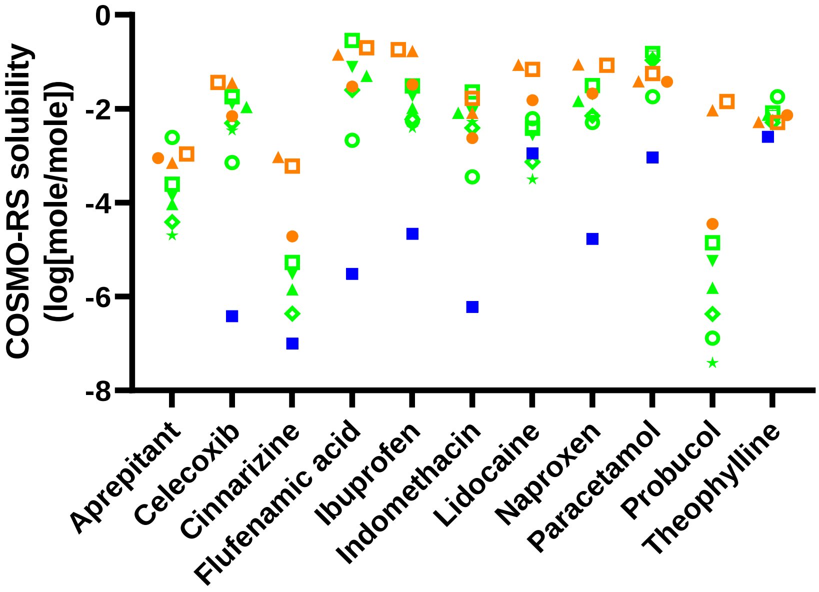

Figure S2. The COSMO-RS predicted solubility of the APIs in ■water, ●glycerol, □ethanol, ▲PEG 300 and the six different DESs (★CU, ▼CG, □BGW, ◇CGluW, ○LGluW, and ▲CLW).

Numeric values for Figure 2-5

Figure 2

	CU	CG	BGW	CGluW	LGluW
Aprepitant	-5.66709	-6.11223	-5.06772	-5.25645	-2.95221
Celecoxib	-3.39342	-2.96034	-3.17124	-3.99557	-3.40879
Cinnarizine	-5.50165	-4.76617	-5.28706	-5.00708	
Flufenamic acid	-3.17029	-3.39584	-2.47514	-4.22359	-4.60309
Ibuprofen	-2.42795	-2.63153	-2.72839	-3.21875	-2.61166
Indomethacin	-2.68867	-2.81787	-2.28731	-3.59692	-2.93309
Lidocaine	-3.29734	-2.57401	-2.9305	-3.32622	-0.63903
Naproxen	-2.44259	-2.68083	-2.79867	-3.11276	-2.98603
Paracetamol	-1.13042	-1.15733	-1.36076	-1.45708	-1.84181
Probucol	-5.27374	-5.36	-4.9909	-5.16586	-5.79567
Theophylline	-2.97722	-3.76854	-2.82352	-3.24116	-1.52285
	CLW	Water	Ethanol	Glycerol	PEG 300
Aprepitant	-3.35898		-3.37047	-3.79684	-3.24828
Celecoxib	-2.24731	-7.15476	-1.87755	-4.09652	-0.46887
Cinnarizine	-3.05859	-7.55348	-2.92167	-5.17534	-1.99494
Flufenamic acid	-2.68732	-6.7486	-1.25109	-3.834	-0.49933
Ibuprofen	-2.10467	-5.316	-0.78573	-2.98252	-0.41047
Indomethacin	-2.28728	-5.55387	-1.61976	-3.46256	-0.2294
Lidocaine		-3.53284	-2.97165	-2.96117	-0.99487
Naproxen	-2.14623	-5.45098	-1.61688	-3.30245	-0.32152
Paracetamol	-0.79573	-3.025	-1.27448	-1.89132	-0.73726
Probucol	-5.13956		-2.48552	-4.26496	-1.95371
Theophylline	-2.58352	-3.19205	-3.68547	-3.07127	-2.68996

Figure 3

	CU	CG	BGW	CGluW	LGluW
Aprepitant	-4.6946	-3.8606	-3.6070	-4.4101	-2.6111
Celecoxib	-2.4591	-1.8904	-1.7443	-2.3099	-3.1485
Cinnarizine	-7.0189	-5.5152	-5.2738	-6.3639	
Flufenamic acid	-1.6033	-1.1080	-0.5423	-1.5994	-2.6697
Ibuprofen	-2.4082	-1.7237	-1.5158	-2.2271	-2.2555
Indomethacin	-2.2822	-2.0717	-1.6405	-2.4055	-3.4512
Lidocaine	-3.5049	-2.5541	-2.4147	-3.1313	-2.2097
Naproxen	-2.1202	-1.6933	-1.5101	-2.1475	-2.2901
Paracetamol	-0.9488	-0.8892	-0.8230	-0.9673	-1.7418
Probucol	-7.4146	-5.2385	-4.8557	-6.3741	-6.8850
Theophylline	-2.1432	-2.1479	-2.0931	-2.2940	-1.7422
	CLW	Water	Ethanol	Glycerol	PEG 300
Aprepitant	-4.0345		-2.9623	-3.0527	-3.1562
Celecoxib	-1.9666	-6.4214	-1.4363	-2.1605	-1.4537
Cinnarizine	-5.8464	-6.9975	-3.2244	-4.7208	-3.0282
Flufenamic acid	-1.3010	-5.5190	-0.7023	-1.5290	-0.8534
Ibuprofen	-1.9874	-4.6613	-0.7388	-1.4884	-0.7730
Indomethacin	-2.0928	-6.2204	-1.7785	-2.6242	-2.0865
Lidocaine		-2.9505	-1.1620	-1.8185	-1.0664
Naproxen	-1.8369	-4.7755	-1.0738	-1.6745	-1.0582
Paracetamol	-0.8992	-3.0406	-1.2488	-1.4231	-1.4204
Probucol	-5.8146		-1.8467	-4.4558	-2.0365
Theophylline	-2.1292	-2.5968	-2.2924	-2.1375	-2.2855

Figure 4

API	DES	Exp. Rel.sol. (LOG[mole/mole])	Rel. Comp. sol. (LOG[mole/mole])	API	DES	Exp. Rel. sol. (LOG[mole/mole])	Rel. comp. sol. (LOG[mole/mole])
Naproxen	CU	-2.4426	-2.1202	Celecoxib	CU	-3.3934	-2.4591
	CG	-2.6808	-1.6933		CG	-2.9603	-1.8904
	CL	-2.7987	-1.5101		CL	-3.1712	-1.7443
	BGW	-3.1128	-2.1475		BGW	-3.9956	-2.3099
	CGW	-2.9860	-2.2901		CGW	-3.4088	-3.1485
	LGluW	-2.1462	-1.8369		LGluW	-2.2473	-1.9666
	Water	-5.4510	-4.7755		Water	-7.1548	-6.4214
	Ethanol	-1.6169	-1.0738		Ethanol	-1.8776	-1.4363
	Glycerol	-3.3025	-1.6745		Glycerol	-4.0965	-2.1605
	PEG 300	-0.3215	-1.0582		PEG 300	-0.4689	-1.4537
Paracetamol	CU	-1.1304	-0.9488	Flufenamic acid	CU	-3.1703	-1.6033
	CG	-1.1573	-0.8892		CG	-3.3958	-1.1080
	CL	-1.3608	-0.8230		CL	-2.4751	-0.5423
	BGW	-1.4571	-0.9673		BGW	-4.2236	-1.5994
	CGW	-1.8418	-1.7418		CGW	-4.6031	-2.6697
	LGluW	-0.7957	-0.8992		LGluW	-2.6873	-1.3010
	Water	-3.0250	-3.0406		Water	-6.7486	-5.5190
	Ethanol	-1.2745	-1.2488		Ethanol	-1.2511	-0.7023
	Glycerol	-1.8913	-1.4231		Glycerol	-3.8340	-1.5290
	PEG 300	-0.7373	-1.4204		PEG 300	-0.4993	-0.8534
Ibuprofen	CU	-2.4279	-2.4082	Cinnarizine	CU	-5.5016	-7.0189
	CG	-2.6315	-1.7237		CG	-4.7662	-5.5152
	CL	-2.7284	-1.5158		CL	-5.2871	-5.2738
	BGW	-3.2188	-2.2271		BGW	-5.0071	-6.3639
	CGW	-2.6117	-2.2555		CGW		
	LGluW	-2.1047	-1.9874		LGluW	-3.0586	-5.8464
	Water	-5.3160	-4.6613		Water	-7.5535	-6.9975
	Ethanol	-0.7857	-0.7388		Ethanol	-2.9217	-3.2244
	Glycerol	-2.9825	-1.4884		Glycerol	-5.1753	-4.7208
	PEG 300	-0.4105	-0.7730		PEG 300	-1.9949	-3.0282
Theophylline	CU	-2.9772	-2.1432	Aprepitant*	CU	-5.6671	-4.6946
	CG	-3.7685	-2.1479		CG	-6.1122	-3.8606
	CL	-2.8235	-2.0931		CL	-5.0677	-3.6070
	BGW	-3.2412	-2.2940		BGW	-5.2565	-4.4101
	CGW	-1.5228	-1.7422		CGW	-2.9522	-2.6111
	LGluW	-2.5835	-2.1292		LGluW	-3.3590	-4.0345
	Water	-3.1921	-2.5968		Water	-6.3064	
	Ethanol	-3.6855	-2.2924		Ethanol	-3.3705	-2.9623
	Glycerol	-3.0713	-2.1375		Glycerol	-3.7968	-3.0527
	PEG 300	-2.6900	-2.2855		PEG 300	-3.2483	-3.1562
Indomethacin	CU	-2.6887	-2.2822	Probucol*	CU	-5.2737	-7.4146
	CG	-2.8179	-2.0717		CG	-5.3600	-5.2385
	CL	-2.2873	-1.6405		CL	-4.9909	-4.8557
	BGW	-3.5969	-2.4055		BGW	-5.1659	-6.3741

	CGW	-2.9331	-3.4512	CGW	-5.7957	-6.8850
	LGluW	-2.2873	-2.0928	LGluW	-5.1396	-5.8146
	Water	-5.5539	-6.2204	Water		
	Ethanol	-1.6198	-1.7785	Ethanol	-2.4855	-1.8467
	Glycerol	-3.4626	-2.6242	Glycerol	-4.2650	-4.4558
	PEG 300	-0.2294	-2.0865	PEG 300	-1.9537	-2.0365
Lidocaine	CU	-3.2973	-3.5049			
	CG	-2.5740	-2.5541			
	CL	-2.9305	-2.4147			
	BGW	-3.3262	-3.1313			
	CGW	-0.6390	-2.2097			
	LGluW					
	Water	-3.5328	-2.9505			
	Ethanol	-2.9716	-1.1620			
	Glycerol	-2.9612	-1.8185			
	PEG 300	-0.9949	-1.0664			

Figure 5

API	DES	Exp. Rel.sol. (LOG[mole/mole])	Rel. Comp. sol. (LOG[mole/mole])	API	DES	Exp. Rel. sol. (LOG[mole/mole])	Rel. comp. sol. (LOG[mole/mole])
Naproxen	CU	3.0084	2.6553	Celecoxib	CU	3.7613	3.9623
	CG	2.7701	3.0821		CG	4.1944	4.5310
	CL	2.6523	3.2654		CL	3.9835	4.6772
	BGW	2.3382	2.6280		BGW	3.1592	4.1115
	CGW	2.4650	2.4854		CGW	3.7460	3.2729
	LGluW	3.3048	2.9386		LGluW	4.9074	4.4548
Paracetamol	CU	1.8946	2.0919	Flufenamic acid	CU	3.5783	3.9157
	CG	1.8677	2.1514		CG	3.3528	4.4110
	CL	1.6642	2.2176		CL	4.2735	4.9767
	BGW	1.5679	2.0733		BGW	2.5250	3.9196
	CGW	1.1832	1.2988		CGW	2.1455	2.8493
	LGluW	2.2293	2.1415		LGluW	4.0613	4.2180
Ibuprofen	CU	2.8881	2.2531	Cinnarizine	CU	2.0518	-0.0213
	CG	2.6845	2.9376		CG	2.7873	1.4823
	CL	2.5876	3.1455		CL	2.2664	1.7238
	BGW	2.0972	2.4342		BGW	2.5464	0.6336
	CGW	2.7043	2.4058		CGW		
	LGluW	3.2113	2.6739		LGluW	4.4949	1.1512
Theophylline	CU	0.2148	0.4536	Aprepitant*	CU	-2.2966	-1.7323
	CG	-0.5765	0.4490		CG	-2.7418	-0.8983
	CL	0.3685	0.5038		CL	-1.6973	-0.6447
	BGW	-0.0491	0.3029		BGW	-1.8860	-1.4478
	CGW	1.6692	0.8546		CGW	0.4183	0.3512
	LGluW	0.6085	0.4676		LGluW	0.0115	-1.0721
Indomethacin	CU	2.8652	3.9382	Probucol*	CU	-2.7882	-5.5680
	CG	2.7360	4.1487		CG	-2.8745	-3.3918
	CL	3.2666	4.5799		CL	-2.5054	-3.0090
	BGW	1.9569	3.8149		BGW	-2.6803	-4.5274
	CGW	2.6208	2.7692		CGW	-3.3101	-5.0383
	LGluW	3.2666	4.1275		LGluW	-2.6540	-3.9679
Lidocaine	CU	0.2355	-0.5544				
	CG	0.9588	0.3963				
	CL	0.6023	0.5358				
	BGW	0.2066	-0.1809				
	CGW	2.8938	0.7407				
